# Glioma Grading on Conventional MR Images: A Deep Learning Study With Transfer Learning

**DOI:** 10.3389/fnins.2018.00804

**Published:** 2018-11-15

**Authors:** Yang Yang, Lin-Feng Yan, Xin Zhang, Yu Han, Hai-Yan Nan, Yu-Chuan Hu, Bo Hu, Song-Lin Yan, Jin Zhang, Dong-Liang Cheng, Xiang-Wei Ge, Guang-Bin Cui, Di Zhao, Wen Wang

**Affiliations:** ^1^Functional and Molecular Imaging Key Lab of Shaanxi Province, Department of Radiology, Tangdu Hospital, Fourth Military Medical University, Xi’an, China; ^2^Computer Network Information Center, Chinese Academy of Sciences, Beijing, China; ^3^Student Brigade, Fourth Military Medical University, Xi’an, China; ^4^Institute of Computing Technology, Chinese Academy of Sciences, Beijing, China

**Keywords:** deep learning, convolutional neural network (CNN), transfer learning, glioma grading, magnetic resonance imaging (MRI)

## Abstract

**Background:** Accurate glioma grading before surgery is of the utmost importance in treatment planning and prognosis prediction. But previous studies on magnetic resonance imaging (MRI) images were not effective enough. According to the remarkable performance of convolutional neural network (CNN) in medical domain, we hypothesized that a deep learning algorithm can achieve high accuracy in distinguishing the World Health Organization (WHO) low grade and high grade gliomas.

**Methods:** One hundred and thirteen glioma patients were retrospectively included. Tumor images were segmented with a rectangular region of interest (ROI), which contained about 80% of the tumor. Then, 20% data were randomly selected and leaved out at patient-level as test dataset. AlexNet and GoogLeNet were both trained from scratch and fine-tuned from models that pre-trained on the large scale natural image database, ImageNet, to magnetic resonance images. The classification task was evaluated with five-fold cross-validation (CV) on patient-level split.

**Results:** The performance measures, including validation accuracy, test accuracy and test area under curve (AUC), averaged from five-fold CV of GoogLeNet which trained from scratch were 0.867, 0.909, and 0.939, respectively. With transfer learning and fine-tuning, better performances were obtained for both AlexNet and GoogLeNet, especially for AlexNet. Meanwhile, GoogLeNet performed better than AlexNet no matter trained from scratch or learned from pre-trained model.

**Conclusion:** In conclusion, we demonstrated that the application of CNN, especially trained with transfer learning and fine-tuning, to preoperative glioma grading improves the performance, compared with either the performance of traditional machine learning method based on hand-crafted features, or even the CNNs trained from scratch.

## Introduction

Glioma is the most common central nervous system tumor, which is classified into World Health Organization (WHO) grades I–IV according to the invasively histopathology results. The preoperative grading of glioma, particularly the differentiation between lower grade glioma (LGG, grades II and III) and higher grade glioma (HGG, grade IV) (Li-Chun Hsieh et al., 2017), is of the utmost importance in treatment planning and prognosis prediction (Wu et al., 2012; Wen and Huse, 2017).

Magnetic resonance imaging (MRI) has become the essential way for glioma diagnosis before surgery. Both conventional and advanced MRI modalities have been analyzed by extracting statistical variances, histogram features or texture features at region of interest (ROI) or pixel level (Liang et al., 2017; Qi et al., 2017; Sharma et al., 2017; Zhao et al., 2017). Although significantly different image features between groups were identified and demonstrated promising sensitivity in glioma grading, it is still far from the accurate personalized diagnosis.

In recent years, machine learning technique has been applied in glioma grading (Wu et al., 2015; Li-Chun Hsieh et al., 2017; Zhang et al., 2017), that the discrimination feature pattern was automatically learned from a set of training data and the corresponding model to predict the individual glioma grade was established afterwards. In these studies, satisfying performances were derived by extracting image features, including clinical features, histogram features and texture features, from varied parameter maps. Different feature normalization and selection algorithms were applied to improve the efficacy of the discrimination model. However, there are two main weaknesses of the traditional machine learning method. First, imaging preprocessing procedure is complex and time-consuming, which highly dependent on the experience of the operators. Second, the robustness of the discrimination model is low. These two factors make the studies be in bench and are far away to bed.

The recent revived technique, deep learning, has shown its potential in the assessment of medical problems, especially convolutional neural network (CNN) (Gulshan et al., 2016; Esteva et al., 2017). It is well-known by its promising robustness and self-learning capability. Instead of extracting features manually, deep CNN automatically learns deeper and abstracted image features during the training procedure (Shen et al., 2017). There have been lots of studies which take advantage of CNN to solve medical problems, such as cancer detection (Ehteshami Bejnordi et al., 2017; Wang et al., 2017) and classification (Chen et al., 2017; Yasaka et al., 2017), and have got excellent performance compared with the methods applied in previous studies. As for glioma, deep learning has shown promising capabilities in predicting key molecular markers such as 1p19q codeletion and MGMT promoter methylation by using MRI images (Akkus et al., 2017; Korfiatis et al., 2017). While, unlike natural images, the major challenge in medical image domain is the insufficient amount of training data.

Transfer learning is an effective method to solve this problem and has been applied and evaluated in several studies (Li et al., 2014; Shin et al., 2016; Tajbakhsh et al., 2016). Deep convolutional activation features learned from the large scale natural image database, ImageNet, had been successfully transferred to the classification and segmentation of histopathology images with little training data (Xu et al., 2017). Meanwhile, ImageNet pre-trained CNNs had been used for detection in X-ray and CT modalities and they had yielded the best performance results (Bar et al., 2015; Ginneken et al., 2015). Yang et al. (2014) pointed out that transfer learning reduced the data distribution mismatch between the training and testing data, which was a main problem for the low accuracy of traditional machine learning method. Thus, we hypothesized that deep learning combined with transfer learning method can achieve high accuracy in distinguishing WHO grade in gliomas.

In this study, we aimed to train a CNN to non-invasively classify LGG and HGG by analyzing on conventional MRI images. First, we explored and evaluated the performance of two CNN architectures (AlexNet and GoogLeNet), with varied parameters and layers in glioma grading. Due to the relatively small sample size, we then evaluated the influence of transfer learning from ImageNet via fine-tuning. All the classification performances were investigated and reported under the patient level five-fold cross-validation.

## Materials and Methods

### Patient Cohort

The study data of the current project derived from a diagnostic trial that has been registered to ClinicalTrials.gov (NCT02622620^1^) with the trial protocol published (Liu et al., 2017). A total of 113 histologically confirmed (Fuller and Scheithauer, 2007) glioma patients were retrospectively enrolled, approved by the Ethic Committee of Tangdu Hospital of the Fourth Military Medical University (TDLL-20151013). Written informed consent was obtained from all individuals. Each participant underwent preoperative conventional and advanced MRI scans on a 3.0T MRI scanner (Discovery 750, GE Healthcare, Milwaukee, WI, United States) with an 8-channel head coil.

The study group comprised 52 patients (grade II: 25, grade III: 27) with LGG and 61 patients with HGG. Number of raw images with tumor in LGG and HGG group was 368 and 499, respectively. The ages of the LGG and HGG cohort range from 10 to 66 years old and 13 to 87 years old, respectively. Lesion location of the 113 glioma patients was summarized according to Vasari MRI Visual Feature Guide in Table 1. Tumors located in frontal lobe, temporal lobe and midline account for most of them.

**Table 1:** Patient baseline characteristics.

Tumor location	Tumor number
Frontal lobe_R	23
Frontal lobe_L	18
Temporal lobe_R	18
Temporal lobe_L	16
Midline	11
Basal ganglia_R	4
Insular lobe_L	3
Parietal lobe_L	3
Bilateral multicentric	2
Cerebellar hemisphere_R	2
Insular lobe_R	2
Occipital lobe_R	2
Parietal lobe_R	2
Bilateral frontal lobe	1
Cerebellar hemisphere_L	1
Lateral ventricles_R	1
Occipital lobe_L	1
Temporo-parietal junction_L	1
Temporo-parietal junction_R	1
Thalamus_R	1


### Data Acquisition

The MR images were obtained on a GE discovery 750 3T MR scanner with an eight-channel head coil. Conventional MRI, including axial pre- and post-contrast T1-weighted imaging (T1-C and T1+C, respectively), pre-contrast T2-weighted imaging (T2WI) and pre-contrast Fluid Attenuated Inversion Recovery (FLAIR) and functional MRI, including dynamic contrast enhanced (DCE) MRI, diffusion weighted imaging (DWI) and arterial spin labeling (ASL), were all obtained. However, up to now, advanced MRI has not been applied in routine clinical diagnosis. In this study, only the most useful sequence, T1+C, was analyzed to make it more convenient for doctors and closer to clinic. The images were acquired using spoiled gradient recalled-echo inversion-recovery prepped (SPGR-IR prepped) sequence (TR/TE = 1750/24 ms, slice thickness = 5 mm, slice spacing = 1.5 mm, field of view (FOV) = 24 × 24 cm^2^, matrix = 256 × 256, number of excitation (NEX) = 1), after the completion of DCE MRI following a total Gadodiamide dosage of 0.2 mmol/kg as described below.

### Image Preprocessing

**FIGURE 1:**
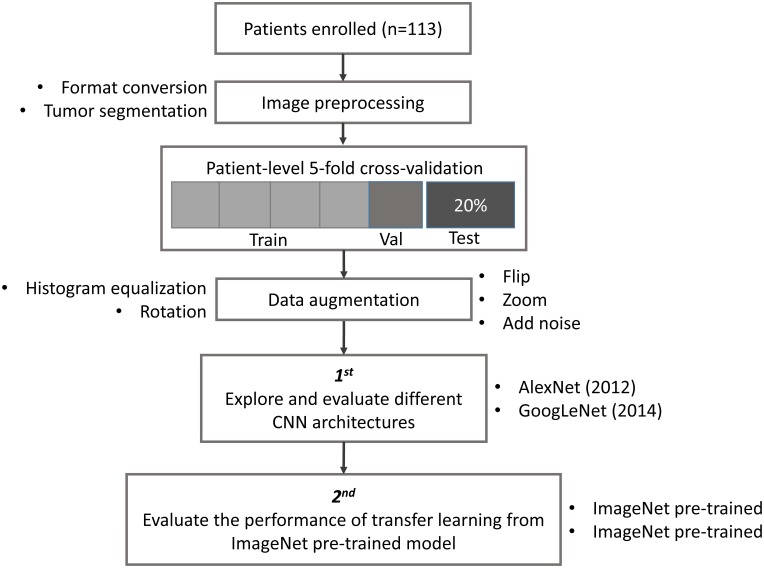
Study design. Patient underwent magnetic resonance imaging (MRI) before surgery. Slices with tumor were selected and segmented as rectangular region of interest (ROI) after images format conversion. Then, data were split into training, validation and test dataset. The data used in five-fold cross-validation were augmented 14 times, and keep the test dataset unchanged. Finally, AlexNet and GoogLeNet were explored and evaluated by training from scratch and fine-tuning from pre-trained model.

The study design, including image preprocessing, data sampling and model training, is shown in Figure 1. In image preprocessing, first, DICOM images were converted into BMP format without annotation. Then, images containing tumor tissue were selected by two experienced neuroradiologists (L Yan and Y Han). The targeted tumor was segmented by a rectangular region of interest (ROI), which contained about 80% area of the tumor area. Then, 20% data were randomly selected and leaved out on patient-level as test dataset, including 10 LGGs and 13 HGGs, which remains unchanged during the experiment. The classification task was evaluated using the rest 80% data with five-fold cross-validation (CV) on patient-level split, in order to avoid that the slices from one subject appear in both training and test data and lead to false positive results. It is more informative for real clinical performance. Data augmentation plays a vital role in the utilization of CNN in medical image, and can efficiently improve the performance. The training data were augmented 14 times by introducing histogram equalization, random rotation, zooming, adding noise (salt and pepper) and flipping (horizontal and vertical) using MATLAB 2016a (Akkus et al., 2017; Chang et al., 2017), and the test data was kept as origin. Afterwards, the prepared data were converted into LMDB format for *caffe*. During this step, the mean image of training set was subtracted for every training and test image, which is recommended in Stanford Tutorial for preprocessing of deep learning.

### Training Protocol

This whole procedure were modified and fine-tuned on the NVIDIA Digits software, which simplifies common deep learning tasks such as managing data, designing and training neural networks on multi-GPU systems, monitoring performance in real time with advanced visualizations, and selecting the best performing model from the results browser for deployment^2^, with CUDA 8.0/cuDNN 5.0 (Nvidia Corporation, Santa Clara, Calif) dependencies for graphics processing unit acceleration. All the experiments were conducted on four NVIDIA Tesla K80 cards.

**FIGURE 2:**
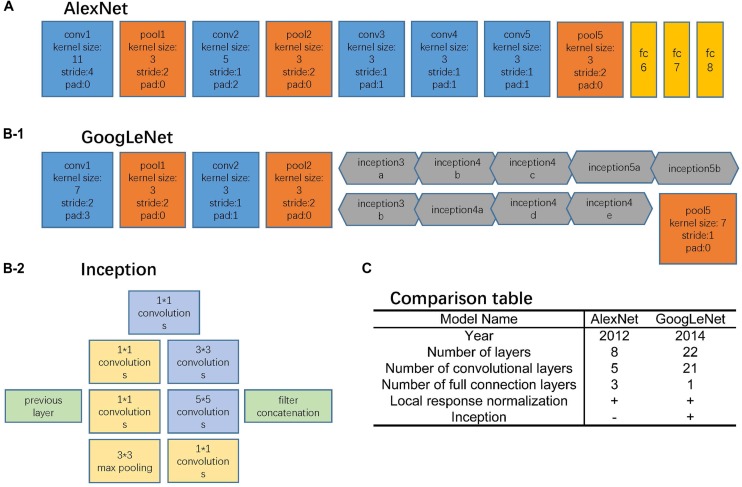
Network architectures. **(A)** AlexNet structure, where the blue blocks indicate convolutional layers, orange blocks indicate pooling layers, and yellow blocks indicate fully-connection layers. **(B-1)** GoogLeNet structure, where the blue blocks indicate convolutional layers, orange blocks indicate pooling layers, and gray blocks indicate inception structure. The specific inception structure was shown in **(B-2)**. **(C)** Comparison table of AlexNet and GoogLeNet.

AlexNet and GoogLeNet can be either trained from scratch or fine-tuned from ImageNet pre-trained models. The comparison between these two architectures is shown in Figure 2. For training from scratch, all the parameters of the models were initialized with random Gaussian distributions and trained for 30 epochs with the mini-batch size of 50 image instances. Training convergence can be observed within 30 epochs. The other hyperparameters are momentum: 0.9; weight decay: 0.0005; base learning rate: 0.01, decreased by a factor of 10 at every 10 epochs. For fine-tuning, the base learning rate was set 10 times smaller than the default learning rate, which was 0.001 (Razavian et al., 2014; Girshick et al., 2016).

#### AlexNet

The AlexNet architecture (Figure 2A) achieved significantly improved performance over the other non-deep learning methods for ImageNet Large Scale Visual Recognition Challenge (ILSVRC) 2012 (Krizhevsky et al., 2012). This success has revived the interest in CNNs in computer vision. It has 60 million parameters and 500,000 neurons, consists of five convolutional layers followed by three fully connected layers and a softmax classifier. The pooling layers were placed after the first, second, and fifth convolutional layers. During the training procedure, images were normalized to 224 pixels × 224 pixels to match the input dimension of the input layer.

#### GoogLeNet

The GoogLeNet model (Figure 2B) is significantly more complex and deep than all previous CNN architectures (Szegedy et al., 2015). More importantly, it also introduces a new module called “Inception” (Figure 2B-2), which concatenates filters of different sizes and dimensions into a single new filter. Overall, GoogLeNet has two convolutional layers, two pooling layers, and nine “Inception” modules. Each “Inception” module consists of six convolution layers and one pooling layer. Although it has 56 convolutional layers in total, only 7 million parameters are included. GoogLeNet is the current state-of-the-art CNN architecture for the ILSVRC, where it achieved 5.5% top-5 classification error on the ImageNet challenge, compared to AlexNet’s 15.3% top-5 classification error. During the training procedure, images were normalized to 256 pixels × 256 pixels to match the input dimension of the input layer.

For both neural networks, the softmax classifier provides a probability for each of the categories for a given input image. The category with the highest predicted probability was taken as the classifier prediction for the image, and we calculated classification accuracy based on this prediction.

#### Transfer Learning

**FIGURE 3:**
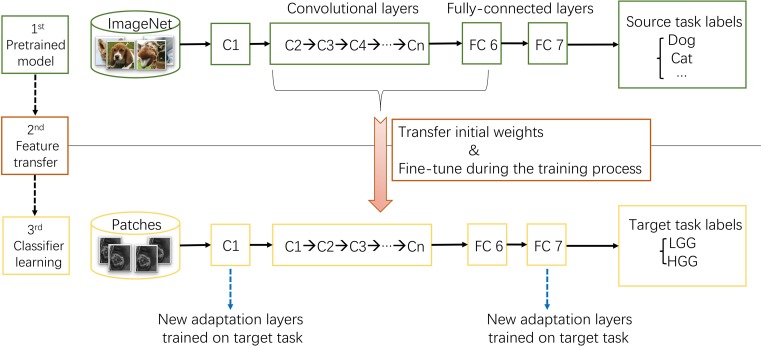
The procedure of transfer learning. First, models trained on ImageNet dataset were downloaded. Second, most parameters of the pre-trained models were transferred to the new target task model. Third, first convolutional layer and fully-connected layers were trained on target task.

ImageNet, which was used in ILSVRC, consists of 1.2 million 256 × 256 × 3 images belonging to 1000 categories (Russakovsky et al., 2015). At times, the objects in the image are small and obscure, and thus pose more challenges for learning a successful classification model. However, the models took the first place have reached satisfactory performance (Krizhevsky et al., 2012; Szegedy et al., 2015). After training from scratch, pre-trained AlexNet and GoogLeNet models were downloaded^3^ and fine-tuned at a learning rate which was 10 times smaller than the default learning rate for the glioma grading task. This procedure was explained in Figure 3. Since the images in ImageNet are three-channel natural images, most of the images in medical domain are one-channel gray images. And there are only two categories need to be exported in our task. Thus, the first convolutional layer and fully-connected layers were randomized initialized and freshly trained, in order to accommodate the new object categories. The initial weights of other layers of the pre-trained model were transferred to the new model directly and fine-tuned during the training process for the new task.

### Model Validation and Test

The performances of models were evaluated by assessing the accuracy on training, validation, and test dataset with five-fold CV on patient level. The classification results of one test image is shown as the probability that the image be classified into each category. Then, the probability of images come from one subject were averaged together to get the classification probability of this subject. The performance measures of each models were analyzed based on the patient-level probability. Meantime, the Area Under Curve (Ehteshami Bejnordi et al., 2017) of Receiver Operator Characteristic (ROC) of test data and cut-off value were calculated using GraphPad Prism Version 6.0. All the performance measures, including accuracy, loss and AUC, were the mean value of five-fold CV.

### Statistical Analysis

The normal distribution and *t*-test were tested to compare the age between patients with LGG and HGG by GraphPad Prism Version 6.0 software. Chi-square and Fisher exact tests were performed to compare the gender between two groups using SPSS version 19.0 software. All differences were considered statistically significant at *p* < 0.05.

## Results

### The Performances of CNNs Trained From Scratch

**FIGURE 4:**
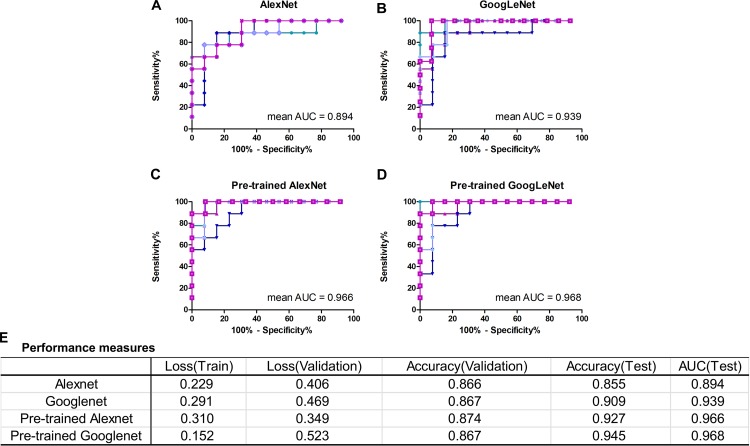
Classification performance of AlexNet and GoogLeNet trained from scratch and fine-tuned from pre-trained model. **(A–D)** ROC curves of data test on five validated models of AlexNet, GoogLeNet, pre-trained AlexNet and pre-trained GoogLeNet, respectively. **(E)** Performance measures table, including training loss, validation loss, validation accuracy, test accuracy and test AUC, of the four models.

In this work, we mainly focused on AlexNet and GoogLeNet. The performance measures averaged for five-fold CV were reported in Figure 4E, including train loss, validation loss, validation accuracy, test accuracy and test AUC. The ROC curves of test data of AlexNet and GoogLeNet of each CV were plotted in Figures 4A,B. As could be seen, GoogLeNet had a better performance than AlexNet. The mean value of validation accuracy, test accuracy and test AUC of GoogLeNet was 0.867, 0.909, and 0.939, respectively. As for AlexNet, the mean value of validation accuracy, test accuracy and test AUC were 0.866, 0.855, and 0.895, respectively. Although GoogLeNet is deeper than AlexNet, it has less parameters owing to the inception structures, which can reduce the risk of overfitting.

### The Influence of Transfer Learning

With transfer learning and fine-tuning, improved performances were obtained for both AlexNet and GoogLeNet. Especially for AlexNet, it reached performances close to GoogLeNet after transfer learning, with a 0.072 increase in test accuracy and AUC. The growth of test accuracy and AUC of GoogLeNet was 0.036 and 0.029. In general, transfer learning is an effective method to improve the classification performance and GoogLeNet performed better than AlexNet. Figures 4C,D shows the ROC curves of these two models after applying transfer learning. Although only T1+C images were used, the test accuracy and AUC reached 0.945 and 0.968 in GoogLeNet (Figure 4E).

**FIGURE 5:**
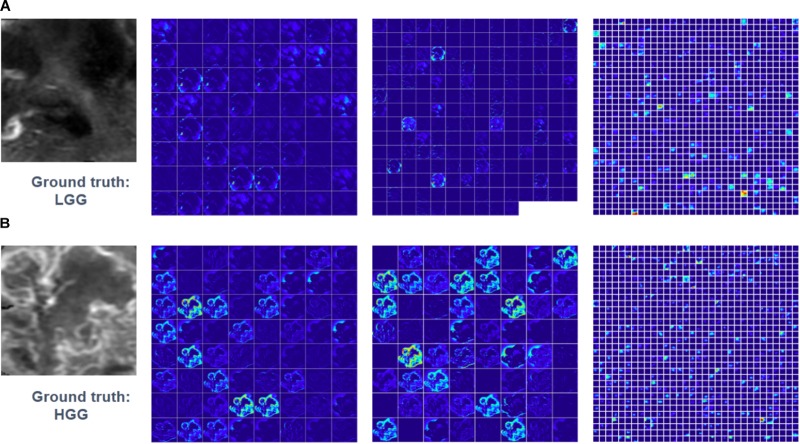
Visualized feature maps of tree convolutional layers in GoogLeNet. **(A)** An example of LGG. **(B)** An example of HGG.

Feature maps of two subjects of three convolutional layers in GoogLeNet were visualized in Figure 5. Normally, feature maps are thought to provide more detailed information as the network become deeper. In the lower convolutional layers, the network only acquires intensity and shape information from the tumor. As the CNN structures become deeper, the features are extracted from feature maps and become more abstract to describe.

**FIGURE 6:**
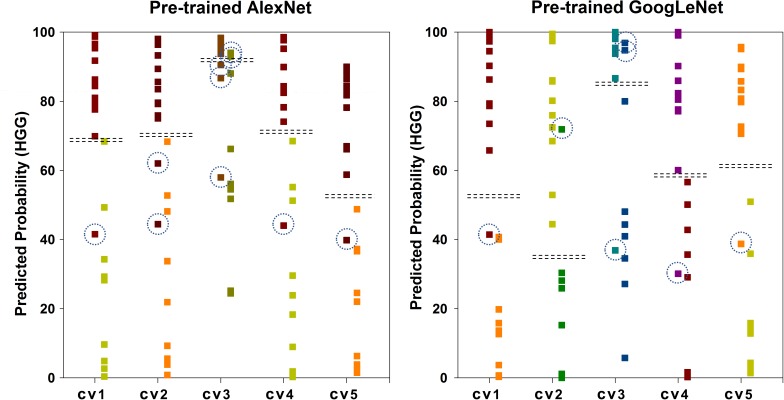
Scatter plot of the mean probability of each subject to be predicted as HGG. The ground truth of the first column of each group is HGG. The ground truth of the second column is LGG. The dash lines in each group indicate the cut-off value of two categories. The dash circles indicate misclassified subjects.

**FIGURE 7:**
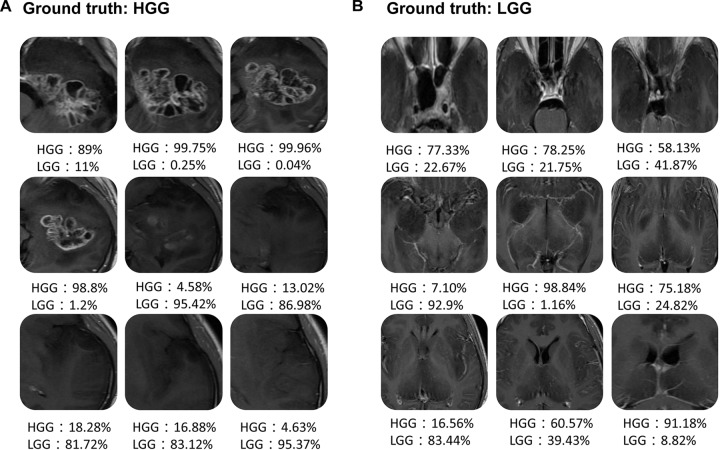
Two examples of the misclassified subjects. **(A)** A HGG located on left temporal lobe, which was classified as LGG. **(B)** A LGG located on midline, which was classified as HGG.

Figure 6 shows the scatter plot of the mean probability of each subject to be predicted as HGG in each cross-validation fold. Although satisfied classification results were got, two subjects among the 22 test subjects were always misclassified. Figure 7 displays the selected slices of these two subjects. The ground truth of Figure 7A is HGG. Due to the non-enhancement of the last five slices, they had high probability to be recognized as LGG. The averaged probability of this subject to be classified into HGG was obviously decreased. Figure 7B was classified into HGG, which should be LGG. This is due to the high signal of cavernous sinus area and choroid plexus on T1WI.

## Discussion

In this study, we demonstrated the utility of deep learning to preoperatively grade glioma by using conventional MRI images. Furthermore, the effect of transfer learning was evaluated on both AlexNet and GoogLeNet. Thus, applications of the fine-tuned CNN image features to glioma grading improves the performance, compared with either the performance-complementary properties of hand-crafted features, or training CNNs from scratch.

Compared with previous studies, improved performances had been got by applying deep learning technology in this study (Zollner et al., 2012; Wu et al., 2015; Qi et al., 2017; Zhang et al., 2017). Previous studies used hand-crafted clinical and/or image features, followed by a complex training procedure. Deep leaning simplified the multi-step pipeline, utilized by learning classification features directly from images, allowing for greater reproducibility. In this study, we demonstrated that accuracy glioma grading can be achieved by without pre-engineered features.

It is observed that AlexNet and GoogLeNet, with 8 and 56 convolutional layers and millions of network parameters, can be useful even in medical domain where the available training data are limited. Not only for the pre-trained model, but also for the models trained from scratch. The required data size is still not sure and is a bottleneck for deep learning studies. Thus, it is crucial to find the trade-off between suitable model and data size (Zhu et al., 2012). Although transfer learning from the ImageNet dataset to medical images has been obvious beneficial in our study, building progressively growing dataset is still important.

Even if natural images and medical images has significant difference, improved precision was got in our study using transfer learning via fine-tuning. In machine learning studies, the basic assumption is that the training data and the future data need to be tested are in the same feature space and have the same distribution. However, the training data for medical classification task is extremely insufficient. It is an effective method to transfer the feature knowledge learned from a large-scale database to a specific medical task (Pan and Yang, 2010; Yosinski et al., 2014). It is known that the features learned from the earliest layers of CNN are usually general features, such as shape, margin and color, which are applicable to many datasets and tasks. The deeper the layer is, the more abstract the learned feature is. Then, the last layer is aim to the specific task. Thus, due to the different dimension of natural RGB images and medical gray images, the first convolutional layer and last fully-connected layer were fine-tuned in our glioma grading task.

Only T1+C MRI sequence was included in our model to grade glioma before surgery. In practical diagnosis, once a mass is identified and hemorrhage is excluded, a contrast-enhanced MRI is typically ordered, with standard T1WI, T2WI, and T2 FLAIR (Cha, 2006; Young, 2007). The volume of various tumor sub-regions (necrotic, enhancing, and non-enhancing), compression of the surrounding tissue and midline deviation can be identified in conventional sequences. T1+C is an essential sequence in routine diagnosis. It is able to indicate the blood-brain barrier breakdown, which is often an indicator of HGG. However, even if advanced MR sequenced have not been used in clinical diagnosis, their potential in glioma grading had been demonstrated in several studies (Qi et al., 2017; Zhang et al., 2017). Dynamic contrast enhanced (DCE) MRI in the preoperative setting measure pharmacokinetic parameters of contrast uptake, which may be associated with early disease progression and survival (Kim et al., 2017). Dynamic susceptibility contrast (DSC) MRI may be helpful in preoperative diagnosis (Chakravorty et al., 2015). Thus, it is necessary to combine advanced MRI sequences to train a radiomics-based deep model for glioma grading in future study.

As illustrated before in Figure 6, there are two main factors that lead to the misclassification. First, since the heterogeneity of glioma, the enhancement condition of each slice in the same tumor has significant difference. Second, the intensity of normal tissue, such as, cavernous sinus area, choroid plexus, and nasal cavity, has obvious influence on the results. To overcome these problems, applying brain extraction technology (BET) before tumor segmentation should be tried in future study. What’s more, combining multi-view images, which are axial, sagittal and coronal view images, may improve the performance.

There are several possible improvements to this study. First and foremost, sufficient cohort size is a limiting factor in the training of deep CNN. Although we overcame this partially by data augmentation and transfer learning technique, a larger patient population would further improve the performance. Second, since the patients were retrospectively enrolled from Jan 2015 to May 2016, the pathology data were not up-to-date with the 2016 WHO classification of gliomas. The IDH status (mutated vs. wildtype) with the histopathology grade should be included in future study. Third, the use of multi-modal and multi-view images, which would provide systemic information of the tumor, may improve the generalizability of the model. Fourth, before the automatically glioma grading, an automatically tumor segmentation model would be necessary to further increase the precision.

## Author Contributions

G-BC, DZ, WW, YY, and L-FY contributed to the conception and design of the study, drafting the work. YY, L-FY, YH, H-YN, Y-CH, BH, S-LY, D-LC, X-WG, XZ, and JZ contributed to the acquisition, the analysis and interpretation of the data, drafting and revision the work. All authors gave the final approval and agreement to all the aspects of the work.

## Conflict of Interest Statement

The authors declare that the research was conducted in the absence of any commercial or financial relationships that could be construed as a potential conflict of interest.

## Footnotes

1 https://www.clinicaltrials.gov/

2 https://developer.nvidia.com/digits

3 https://github.com/NervanaSystems/ModelZoo
